# Supplementary material for: Bayesian learning for the robust verification of autonomous robots
Source: Commun Eng. 2024 Jan 27;3:18. doi: 10.1038/s44172-024-00162-y (PMC11031605; doi:10.1038/s44172-024-00162-y)
Supplement: Supplementary file 1 — Supplementary Material [file 44172_2024_162_MOESM1_ESM.pdf]

Supplementary Material: Bayesian Learning for the Robust Verification of Autonomous Robots

Xingyu Zhao[1], Simos Gerasimou[2], Radu Calinescu[2], Calum Imrie[2], Valentin Robu[3,4], and David Flynn[5]

[1]Warwick Manufacturing Group, University of Warwick, Coventry, UK.
[2]Department of Computer Science, University of York, York, UK
[3]Intelligent and Autonomous Systems Group, Centrum Wiskunde & Informatica, Amsterdam, NL.
[4]Electrical Engineering Department, Eindhoven University of Technology, Eindhoven, NL
[5]James Watt School of Engineering, University of Glasgow, Glasgow, UK.

This supplementary material document includes:

- The proofs to **Corollary 1** and **Corollary 2** from the main paper.
- Details of the experimental settings for the offshore infrastructure maintenance case study from the Results Section of the main paper.

Supplementary Methods 1: Corollary Proofs

Corollary 1. *When $m = 3$, the bounds (7) and (8) in **Theorem 1** of the main paper satisfy:*

$$\lambda_l \geq \begin{cases} \frac{\epsilon_1 l(\epsilon_1)\theta_2}{\theta_1 + l(\epsilon_1)\theta_2}, & \text{if } \frac{\theta_2(\epsilon_1-\epsilon_2)}{\theta_1} > \frac{\epsilon_2 l(\epsilon_2) - \epsilon_1 l(\epsilon_1)}{l(\epsilon_1) l(\epsilon_2)} \\ \frac{\epsilon_2 l(\epsilon_2)\theta_2}{\theta_1 + l(\epsilon_2)\theta_2}, & \text{otherwise} \end{cases} \tag{S1}$$

and

$$\lambda_u < \begin{cases} \frac{\epsilon_1 l(\epsilon_1)\theta_1 + \epsilon_2 l(\epsilon_2)\theta_2 + \frac{1}{t} l(\frac{1}{t})(1-\theta_1-\theta_2)}{l(\epsilon_1)\theta_1}, & \text{if } t < \frac{1}{\epsilon_2} \\ \frac{\epsilon_1 l(\epsilon_1)\theta_1 + \frac{1}{t} l(\frac{1}{t})\theta_2 + \epsilon_2 l(\epsilon_2)(1-\theta_1-\theta_2)}{l(\epsilon_1)\theta_1}, & \text{if } \frac{1}{\epsilon_2} \leq t \leq \frac{1}{\epsilon_1} \\ \frac{\epsilon_1 l(\epsilon_1)(\theta_1+\theta_2) + \epsilon_2 l(\epsilon_2)(1-\theta_1-\theta_2)}{l(\epsilon_1)\theta_1}, & \text{otherwise} \end{cases} \tag{S2}$$

Proof. When $m = 3$, Eq. (8) of **Theorem 1** says, there is a supremum $\lambda_{u,m=3}$:

$$\lambda_{u,m=3} = \max_{\{0 \leq \lambda_1 \leq \epsilon_1 < \lambda_2 \leq \epsilon_2 < \lambda_3 < +\infty\}} \frac{\lambda_1 l(\lambda_1)\theta_1 + \lambda_2 l(\lambda_2)\theta_2 + \lambda_3 l(\lambda_3)(1-\theta_1-\theta_2)}{l(\lambda_1)\theta_1 + l(\lambda_2)\theta_2 + l(\lambda_3)(1-\theta_1-\theta_2)} \tag{S3}$$

Similarly, Eq. (7) of **Theorem 1** shows, when $m = 3$, there is an infimum $\lambda_{l,m=3}$:

$$\lambda_{l,m=3} = \min_{\{0 \leq x_i \leq 1, \forall i \in [1..3]\}} \frac{\sum\limits_{i=1..3} [\epsilon_i l(\epsilon_i)(1-x_i)\theta_i + \epsilon_{i-1} l(\epsilon_{i-1}) x_i \theta_i]}{\sum\limits_{i=1..3} [l(\epsilon_i)(1-x_i)\theta_i + l(\epsilon_{i-1}) x_i \theta_i]} \tag{S4}$$

where $\epsilon_0 = 0$ and $\epsilon_3 = +\infty$ (and thus $l(\epsilon_0) = 1$, $\lim\limits_{\epsilon_3 \to +\infty} l(\epsilon_3) = 0$ and $\lim\limits_{\epsilon_3 \to +\infty} \epsilon_3 l(\epsilon_3) = 0$).

First, we prove the result of (S2). By taking the partial derivative of the objective function in (S3) w.r.t. λ_1, we know the derivative is always positive, irrespective of the values λ_2 and λ_3 take in their respective ranges, as shown below (note $0 \leq \lambda_1 \leq \epsilon_1 < \lambda_2 \leq \epsilon_2 < \lambda_3 < +\infty$):

$$\frac{\partial \frac{\lambda_1 l(\lambda_1)\theta_1 + \lambda_2 l(\lambda_2)\theta_2 + \lambda_3 l(\lambda_3)(1-\theta_1-\theta_2)}{l(\lambda_1)\theta_1 + l(\lambda_2)\theta_2 + l(\lambda_3)(1-\theta_1-\theta_2)}}{\partial \lambda_1} =$$

$$\frac{e^{-\lambda_1 t}\theta_1 \left[e^{-\lambda_1 t}\theta_1 + e^{-\lambda_2 t}\theta_2 \left(1 - (\lambda_1 - \lambda_2)t\right) + e^{-\lambda_3 t}(1-\theta_1-\theta_2)(1-(\lambda_1-\lambda_3)t)\right]}{(e^{-\lambda_1 t}\theta_1 + e^{-\lambda_2 t}\theta_2 + e^{-\lambda_3 t}(1-\theta_1-\theta_2))^2} > 0 \tag{S5}$$

This implies that the maximum point lies in the hyperplane of $\lambda_1 = \epsilon_1$. Thus, we substitute $\lambda_1 = \epsilon_1$ into (S3) and reduce the problem to:

$$\lambda_{u,m=3} = \max_{\{\epsilon_1<\lambda_2\leq\epsilon_2<\lambda_3<+\infty\}} \frac{\epsilon_1 l(\epsilon_1)\theta_1 + \lambda_2 l(\lambda_2)\theta_2 + \lambda_3 l(\lambda_3)(1-\theta_1-\theta_2)}{l(\epsilon_1)\theta_1 + l(\lambda_2)\theta_2 + l(\lambda_3)(1-\theta_1-\theta_2)} \tag{S6}$$

$$< \max_{\{\epsilon_1<\lambda_2\leq\epsilon_2<\lambda_3<+\infty\}} \frac{\epsilon_1 l(\epsilon_1)\theta_1 + \lambda_2 l(\lambda_2)\theta_2 + \lambda_3 l(\lambda_3)(1-\theta_1-\theta_2)}{l(\epsilon_1)\theta_1} \tag{S7}$$

$$\leq \begin{cases} \frac{\epsilon_1 l(\epsilon_1)\theta_1 + \epsilon_2 l(\epsilon_2)\theta_2 + \frac{1}{t} l(\frac{1}{t})(1-\theta_1-\theta_2)}{l(\epsilon_1)\theta_1} & t < \frac{1}{\epsilon_2} \\ \frac{\epsilon_1 l(\epsilon_1)\theta_1 + \frac{1}{t} l(\frac{1}{t})\theta_2 + \epsilon_2 l(\epsilon_2)(1-\theta_1-\theta_2)}{l(\epsilon_1)\theta_1} & \frac{1}{\epsilon_2} \leq t \leq \frac{1}{\epsilon_1} \\ \frac{\epsilon_1 l(\epsilon_1)(\theta_1+\theta_2) + \epsilon_2 l(\epsilon_2)(1-\theta_1-\theta_2)}{l(\epsilon_1)\theta_1} & t > \frac{1}{\epsilon_1} \end{cases} \tag{S8}$$

where the last step is due to the fact that the function $xl(x)$ is unimodal over $[0,1]$ with a maximum point at $x = \frac{1}{t}$. Thus, the last step says:

- When $t < \frac{1}{\epsilon_2}$ (i.e. $\epsilon_2 < \frac{1}{t}$): the function $\lambda_3 l(\lambda_3)$ can reach its maximum at $\lambda_3 = \frac{1}{t}$ in the range $(\epsilon_2, +\infty)$; While, since $\lambda_2 \in (\epsilon_1, \epsilon_2]$, the function $\lambda_2 l(\lambda_2)$ cannot reach $\lambda_2 = \frac{1}{t}$, so we set $\lambda_2 = \epsilon_2$ to maximise the objective function.
- When $\frac{1}{\epsilon_2} \leq t \leq \frac{1}{\epsilon_1}$ (i.e. $\epsilon_1 \leq \frac{1}{t} \leq \epsilon_2$): the function $\lambda_2 l(\lambda_2)$ can attain its maximum at $\lambda_2 = \frac{1}{t}$ in the range $(\epsilon_1, \epsilon_2]$; While, since $\lambda_3 \in (\epsilon_2, +\infty]$, the function $\lambda_3 l(\lambda_3)$ cannot reach $\lambda_3 = \frac{1}{t}$, so we set $\lambda_3 = \epsilon_2$ to maximise the objective function.
- When $t > \frac{1}{\epsilon_1}$ (i.e. $\frac{1}{t} < \epsilon_1$) both the functions $\lambda_3 l(\lambda_3)$ $\lambda_2 l(\lambda_2)$ take the left endpoints in their range to maximise the objective function, so we set $\lambda_3 = \epsilon_2$ and $\lambda_2 = \epsilon_1$.

Substitute the values of λ_2 and λ_3 into the objective function in those three cases, we obtain the results of (S2).

Now we prove the result of (S1)**.** If we denote the objective function in (S4) as a fraction $\frac{Nu(x_1,x_2,x_3)}{De(x_1,x_2,x_3)}$, then take its partial derivative w.r.t. x_3:

$$\frac{\partial \frac{Nu(x_1,x_2,x_3)}{De(x_1,x_2,x_3)}}{\partial x_3} = \frac{l(\epsilon_2)(1-\theta_1-\theta_2)[((1-x_1)\theta_1 + x_2\theta_2)(\epsilon_2-\epsilon_1)l(\epsilon_1) + \epsilon_2 x_1\theta_1]}{De(x_1,x_2,x_3)^2} > 0 \tag{S9}$$

Thus to minimise the objective function, we set $x_3 = 0$. Then we take its partial derivative w.r.t. x_1:

$$\frac{\partial \frac{Nu(x_1,x_2,0)}{De(x_1,x_2,0)}}{\partial x_1} = \frac{-\theta_1[\epsilon_1 l(\epsilon_1)De(x_1,x_2,0) + (1-l(\epsilon_1))Nu(x_1,x_2,0)]}{De(x_1,x_2,0)^2} < 0 \tag{S10}$$

Thus to minimise the objective function, we set $x_1 = 1$. Now we take its partial derivative w.r.t. x_2:

$$\frac{\partial \frac{Nu(1,x_2,0)}{De(1,x_2,0)}}{\partial x_2} = \frac{\theta_2[\theta_2(\epsilon_1-\epsilon_2)l(\epsilon_1)l(\epsilon_2) + \theta_1\epsilon_1 l(\epsilon_1) - \theta_1\epsilon_2 l(\epsilon_2)]}{De(1,x_2,0)^2} \tag{S11}$$

whose sign is determined by other model parameters. Thus, we set $x_2 = \mathbf{1}_{\theta_2(\epsilon_1-\epsilon_2)l(\epsilon_1)l(\epsilon_2)+\theta_1\epsilon_1 l(\epsilon_1)-\theta_1\epsilon_2 l(\epsilon_2)<0}$ where $\mathbf{1}_{\mathtt{S}}$ is an indicator function – it equals 1 when predicate $\mathtt{S}$ is true, and 0 otherwise.

Substitute $x_1 = 1, x_3 = 0$ and $x_2 = \mathbf{1}_{\theta_2(\epsilon_1-\epsilon_2)l(\epsilon_1)l(\epsilon_2)+\theta_1\epsilon_1 l(\epsilon_1)-\theta_1\epsilon_2 l(\epsilon_2)<0}$ into $\frac{Nu(x_1,x_2,x_3)}{De(x_1,x_2,x_3)}$, we obtain two cases in (S1).

□

Corollary 2. *The closed-form BIPP bounds for $m = 2$ can be obtained respectively by setting $\epsilon_2 = \epsilon_1$ and $\theta_2 = 0$ in the results* (S1) *and* (S2)*.*

Proof. When $m = 2$, Eq. (8) of **Theorem 1** becomes the supremum $\lambda_{u,m=2}$ such that (note, $\theta_2 = 1 - \theta_1$):

$$\lambda_{u,m=2} = \max_{\{0\leq\lambda_1\leq\epsilon_1<\lambda_2<+\infty\}} \frac{\lambda_1 l(\lambda_1)\theta_1 + \lambda_2 l(\lambda_2)(1-\theta_1)}{l(\lambda_1)\theta_1 + l(\lambda_2)(1-\theta_1)} \tag{S12}$$

Similarly, Eq. (7) of **Theorem 1** becomes the infimum $\lambda_{l,m=2}$:

$$\lambda_{l,m=2} = \min_{\{0\leq x_1\leq 1, 0\leq x_2\leq 1\}} \frac{\epsilon_0 l(\epsilon_0)x_1\theta_1 + \epsilon_1 l(\epsilon_1)(1-x_1)\theta_1 + \epsilon_1 l(\epsilon_1)x_2(1-\theta_1) + \epsilon_2 l(\epsilon_2)(1-x_2)(1-\theta_1)}{l(\epsilon_0)x_1\theta_1 + l(\epsilon_1)(1-x_1)\theta_1 + l(\epsilon_1)x_2(1-\theta_1) + l(\epsilon_2)(1-x_2)(1-\theta_1)} \tag{S13}$$

where $\epsilon_0 = 0$ and $\epsilon_2 = +\infty$.

First, we prove the bound $\lambda_{u,m=2}$ satisfies:

$$\lambda_{u,m=2} < \begin{cases} \frac{\epsilon_1 l(\epsilon_1)\theta_1 + \frac{1}{t} l(\frac{1}{t})(1-\theta_1)}{l(\epsilon_1)\theta_1} & t < \frac{1}{\epsilon_1} \\ \frac{\epsilon_1}{\theta_1} & t \geq \frac{1}{\epsilon_1} \end{cases} \tag{S14}$$

for which we proceed in two steps:

1. We show the optimised point in the two dimensional space of λ_1 and λ_2 must lie in the plane of $\lambda_1 = \epsilon_1$.
2. In the plane of $\lambda_1 = \epsilon_1$, a closed-form expression can be derived from the monotonicity analysis of λ_2.

By taking the partial derivative of the objective function in (S12) w.r.t. λ_1, we know the derivative is always positive, irrespective of the value take λ_2 in its respective range, as shown in (S15) below (note, $0 \leq \lambda_1 \leq \epsilon_1 < \lambda_2 < +\infty$):

$$\frac{\partial \frac{\lambda_1 e^{-\lambda_1 t}\theta_1 + \lambda_2 e^{-\lambda_2 t}(1-\theta_1)}{e^{-\lambda_1 t}\theta_1 + e^{-\lambda_2 t}(1-\theta_1)}}{\partial \lambda_1} = \frac{e^{-\lambda_1 t}\theta_1 \left[e^{-\lambda_1 t}\theta_1 + e^{-\lambda_2 t}(1-\theta_1)(1-(\lambda_1-\lambda_2)t)\right]}{(e^{-\lambda_1 t}\theta_1 + e^{-\lambda_2 t}(1-\theta_1))^2} > 0 \tag{S15}$$

This implies that the maximum point lies in the plane of $\lambda_1 = \epsilon_1$. Now we reduce the optimisation problem from a two-dimensional space to the one-dimensional space of λ_2. Thus, by substituting $\lambda_1 = \epsilon_1$ in to the r.h.s. of (S12), we have:

$$\begin{aligned} \lambda_{u,m=2} &\leq \max_{\{\lambda_2 > \epsilon_1\}} \frac{\epsilon_1 l(\epsilon_1)\theta_1 + \lambda_2 l(\lambda_2)(1-\theta_1)}{l(\epsilon_1)\theta_1 + l(\lambda_2)(1-\theta_1)} \\ &< \max_{\{\lambda_2 > \epsilon_1\}} \frac{\epsilon_1 l(\epsilon_1)\theta_1 + \lambda_2 l(\lambda_2)(1-\theta_1)}{l(\epsilon_1)\theta_1} \\ &< \begin{cases} \frac{\epsilon_1 l(\epsilon_1)\theta_1 + \frac{1}{t} l(\frac{1}{t})(1-\theta_1)}{l(\epsilon_1)\theta_1} & t < \frac{1}{\epsilon_1} \\ \frac{\epsilon_1}{\theta_1} & t \geq \frac{1}{\epsilon_1} \end{cases} \end{aligned} \tag{S16}$$

where the last step of (S16) is because of the monotonicity analysis of the term $\lambda_2 l(\lambda_2)$ as follows. Depends on the the observable t:

- When $\epsilon_1 < \frac{1}{t}$, $\lambda_2 l(\lambda_2)$ attains its maximum at the critical point $\lambda_2 = \frac{1}{t}$, in the range $\lambda_2 > \epsilon_1$. Thus, we substitute $\lambda_2 = \frac{1}{t}$ and obtain the first case in result (S16).
- When $\epsilon_1 \geq \frac{1}{t}$, in the range $\lambda_2 > \epsilon_1$, we know the supremum of $\lambda_2 l(\lambda_2)$ is attained at the boundary point $\lambda_2 = \epsilon_1$. Thus, we substitute $\lambda_2 = \epsilon_1$ and obtain the second case in result (S16).

Second, we prove the infimum $\lambda_{l,m=2} = 0$ with the optimal point at $x_1 = 1, x_2 = 0$. Since $l(0) = 1$, $\lim_{\epsilon_2 \to +\infty} l(\epsilon_2) = 0$ and $\lim_{\epsilon_2 \to +\infty} \epsilon_2 l(\epsilon_2) = 0$, (S13) can be rewritten as:

$$\lambda_{l,m=2} = \min_{\{0 \leq x_1 \leq 1, 0 \leq x_2 \leq 1\}} \frac{\epsilon_1 l(\epsilon_1)(1-x_1)\theta_1 + \epsilon_1 l(\epsilon_1) x_2 (1-\theta_1)}{x_1\theta_1 + l(\epsilon_1)(1-x_1)\theta_1 + l(\epsilon_1)x_2(1-\theta_1)} \tag{S17}$$

The partial derivative of the objective function in (S17) w.r.t. x_2 is:

$$\frac{\partial \frac{\epsilon_1 l(\epsilon_1)(1-x_1)\theta_1 + \epsilon_1 l(\epsilon_1)x_2(1-\theta_1)}{x_1\theta_1 + l(\epsilon_1)(1-x_1)\theta_1 + l(\epsilon_1)x_2(1-\theta_1)}}{\partial x_2} = \frac{\epsilon_1 l(\epsilon_1)(1-\theta_1)\theta_1 x_1}{[((x_1+x_2-1)\theta_1 - x_2)l(\epsilon_1) - \theta_1 x_1]^2} > 0 \tag{S18}$$

Thus we set $x_2 = 0$ in (S17) to reduce the problem to:

$$\lambda_{l,m=2} = \min_{\{0 \leq x_1 \leq 1\}} \frac{\epsilon_1 l(\epsilon_1)(1-x_1)\theta_1}{x_1\theta_1 + l(\epsilon_1)(1-x_1)\theta_1} \tag{S19}$$

The partial derivative of the objective function in (S19) w.r.t. x_1 is:

$$\frac{\partial \frac{\epsilon_1 l(\epsilon_1)(1-x_1)\theta_1}{x_1\theta_1 + l(\epsilon_1)(1-x_1)\theta_1}}{\partial x_1} = \frac{-\epsilon_1 l(\epsilon_1)}{[x_1 + (1-x_1)l(\epsilon_1)]^2} < 0 \tag{S20}$$

Thus we set $x_1 = 1$ in (S19), and obtain $\lambda_{l,m=2} = 0$. Note, the result of 0 is attainable meaning we cannot find a lower bound that bigger than 0 for the given optimisation problem.

Finally, substitute $\epsilon_2 = \epsilon_1$ and $\theta_2 = 0$ in the results (S2) and (S1), we obtain the results of (S14) and 0 which are the closed-form BIPP bounds for $m = 2$.

□

Supplementary Methods 2: Offshore Infrastructure Maintenance Experiments

Simulation Platform

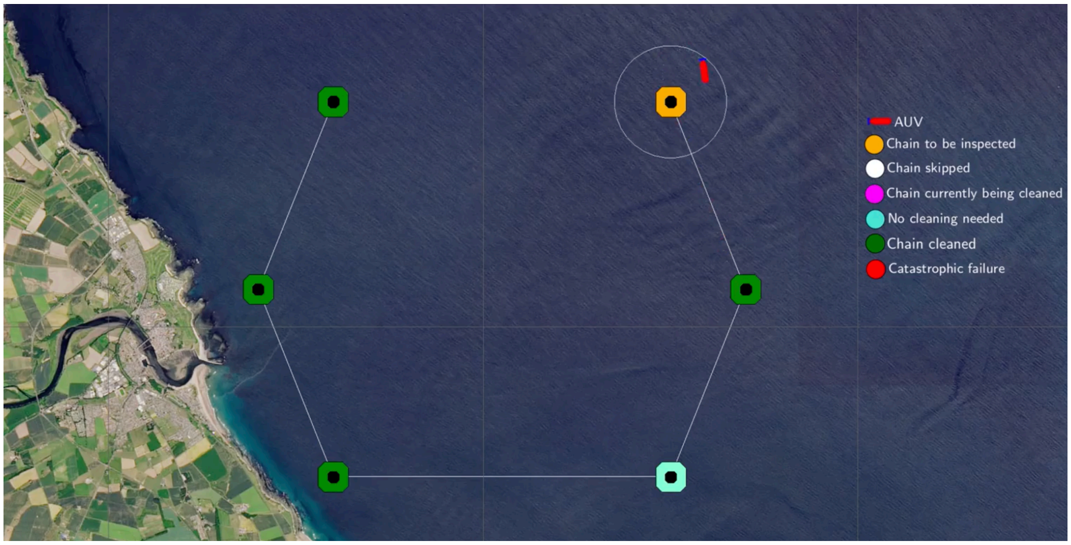

Supplementary figure 1: Illustration of our robust Bayesian verification framework for the structural health inspection and cleaning mission using an autonomous underwater vehicle (AUV) at the point when the AUV inspects the final floating chain.

In the Results Section of the main paper, we demonstrate the application of our robust Bayesian verification framework using a case study that involves an autonomous underwater vehicle (AUV) executing a structural health inspection and cleaning mission of the substructure of an offshore wind farm. The offshore wind farm consists of multiple floating wind turbines. Each turbine is a buoyant foundation structure secured to the sea bed with floating chains tethered to anchors. The AUV is deployed to collect data about the condition of the floating chains to enable the post-mission identification of problems that could affect the structural integrity of the chains. Supplementary figure 1 shows the AUV during the inspection of the last floating chain.

The AUV-based mission is built on top of the open-source framework MOOS-IvP (`http://www.moos-ivp.org`), a widely used platform for the implementation of autonomous applications with AUVs. When used for the execution of oceanic missions, MOOS-IvP is deployed on the payload computer of an AUV, facilitating the decoupling of the vehicle's autonomy from the navigation and control system running on the main AUV computer [1].

An AUV-based system leveraging MOOS-IvP is structured as a community of independent applications running in parallel that communicate via a MOOS database (MOOSDB) using a publish-subscribe architecture. Supplementary figure 2 shows the high-level architecture of MOOS-IvP. Applications publish messages in the form of key-value pairs with specified frequencies, sharing information about AUV components that an application monitors. Interested listening applications can use the keys to subscribe to messages and receive a notification when an update of that message becomes available.

The autonomous operation in MOOV-IvP is instrumented through a collection of behaviours, i.e., combinations of boolean logic constraints and piecewise-linear utility functions parametrised, for example, with parameters of the navigation and control system such as heading, speed or depth. During mission execution, the IvP Helm, the decision-making component of MOOS-IvP, periodically collects and reconciles the instantiated behaviours. If multiple behaviours are active simultaneously, the IvP Helm executes Interval Programming (IvP) multi-objective optimisation to determine the optimal action, i.e., an optimal point in the decision space defined by the constraints and utility functions. This optimal action is expressed as a set of key–value pairs and is published to the MOOSDB so that interested (subscribing) applications can receive this update and act upon it.

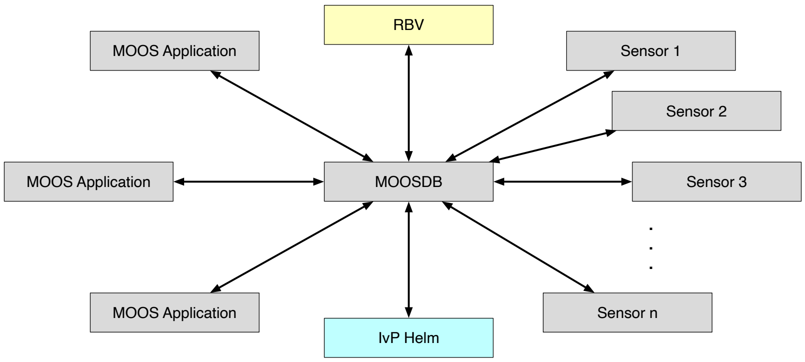

Supplementary figure 2: High-level MOOS-IvP architecture with the RBV framework implementation

To realise the AUV-based floating chain inspection and maintenance mission, we extended the MOOS-IvP framework and developed a new MOOS application (called RBV in supplementary figure 2) that implements the overall mission scenario and controls the mission execution. In particular, the RBV application employs the built-in behaviours MOOS-IvP (e.g., waypoint and station keep) to model the AUV mission and leverages the starting and ending condition of these behaviours to instrument the decision-making via the IvP Helm. Furthermore, the RBV application provides several configuration parameters that enable the execution of custom experiments. For instance, users can define the probabilities and rates characterising the behaviour of each chain (i.e., specialising the continuous-time Markov chain – CTMC, model in the main paper), thus, affecting the UAV behaviour. Using a seed as a configuration parameter enables to reduce the non-determinism of the simulator, thus enhancing the reproducibility of the experiments and the robustness of the results obtained.

The open-source RBV source code, the full experimental results, additional information about RBV, including a video of the floating chain inspection and maintenance mission, are available at `https://github.com/gerasimou/RBV`.

Experimental Methodology

We evaluated the capabilities of our RBV framework by performing a wide range of experiments that assess both the decision support offered by the framework and its overheads. Accordingly, we instrumented the simulation platform (as described in the Section of Simulation Platform in this document) with the implemented RBV framework (main paper, Figure 1) and realised the AUV-driven structural health inspection and cleaning mission presented in the Results Section of the main paper. Given the parametric CTMC model of the mission (main paper, Figure 2), we consider as unknown parameters the chain-dependent transition rate for cleaning the i-th chain (r_i^{clean}), and the mission-dependent transition rates for causing catastrophic damage to a floating chain or itself (r^{damage}) and for failing to clean (r^{fail}). Since the floating chains are spatially located in the same area, we model the failure rate r^{fail} as a homogeneous parameter affecting all chains of the mission similarly. Nevertheless, our RBV framework can be easily adapted to support modelling an individual transition rate for failing to clean (r_i^{fail}) each i-th chain.

We assemble the interval CTMC model using the BIPP and IPSP estimators to learn these unknown model parameters. In particular, we use the BIPP estimator to quantify the rate values associated with the singular events of cleaning the i-th chain (r_i^{clean}) and encountering a catastrophic failure (r^{damage}). The former corresponds to successfully completing a difficult one-off task, and the latter models a major failure. Since the AUV may try multiple times to clean a particular chain, we model the corresponding transition rate (r^{fail}) using the IPSP estimator, which is suitable for events observed regularly during system operation.

Results

We have already presented how our RBV framework supports the runtime verification of mission-critical autonomous robots for a typical scenario of the AUV-based offshore wind-turbine inspection and maintenance mission (main paper, Figure 3). We also measured the overheads associated with executing the online verification process (main paper, Figure 4). Furthermore, we systematically analysed the operation of both BIPP and IPSP estimators in several scenarios with varying levels of partial prior knowledge (main paper, Figures 5 and 6).

In this section, we present additional results for the end-to-end application of the RBV framework, focusing on the AUV behaviour over multiple failed attempts to clean a specific chain. Supplementary figure 3 shows the verification results for requirements R1 – quantifying the probability of the mission completing successfully (top)

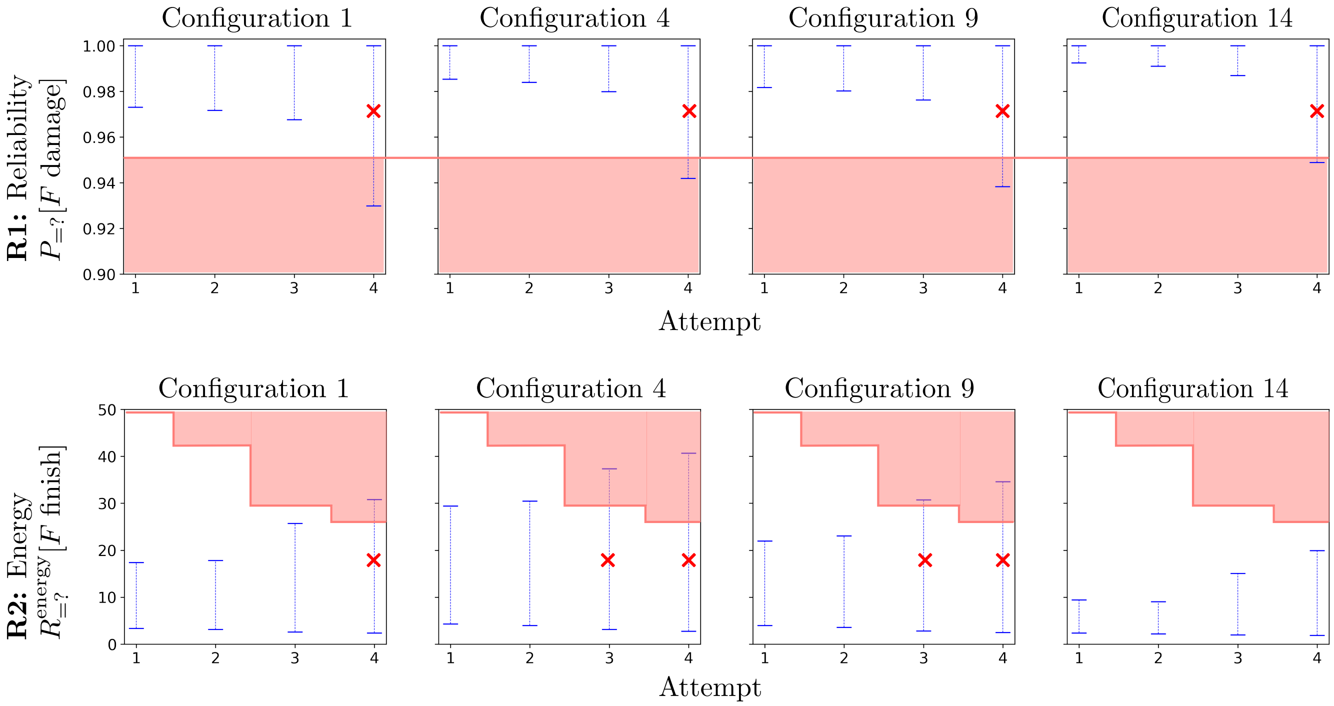

Supplementary figure 3: Computed value intervals for the reliability requirement R1, the probability that the AUV will not encounter a catastrophic failure during its mission (top) and energy requirement R2, the expected energy consumption (bottom), over successive attempts for the same AUV configuration. After a failed attempt, each new attempt for the same chain and AUV configuration results in a wider interval for the key system requirements R1 and R2.

and R2 – quantifying the expected energy consumption of the AUV (bottom) across successive attempts for the same AUV configuration. In each of these plots and irrespective of the system property measured, the computed value intervals become wider as the number of failed AUV attempts to clean the chain increases. For instance, consider requirement R1 and configuration 1 (shown on the top left in supplementary figure 3), which shows a small increase in the reliability interval for the three initial attempts to clean the chain. Despite the interval becoming wider, the reliability threshold of 0.95 is satisfied; thus, this configuration is feasible and is included in the candidates set for further analysis using requirement R3 – selecting the configuration that maximises the number of chains cleaned. In contrast, the computed reliability interval for the fourth attempt violates the reliability threshold; thus, this configuration is infeasible. No valid configuration exists in the fourth attempt, and the AUV decides to skip the chain and move to the next.

A similar pattern of wider value intervals is also observed for the energy consumption property (R2). In this case, the energy threshold decreases for each new attempt as the AUV has consumed energy trying to clean the chain in the previous attempts. Consequently, this requirement is more restrictive and leads to excluding further configurations; see, for instance, the violated energy threshold in attempt 3 for configurations 4 and 9.

The wider intervals over each successive failed attempt correspond to the increased uncertainty concerning the AUV's operation and its capacity to fulfil the mission successfully. The rationale underpinning this behaviour is that since both transition rates r_i^{clean} and r^{damage} employ the BIPP estimator, the posterior estimate bounds for both transition rates are wider and converge towards their theoretical asymptotic values (cf. the section of "BIPP estimator evaluation" in the main paper). However, since the prior knowledge for the r_i^{clean} rate is higher than the r^{damage} rate, the posterior bounds for the r_i^{clean} rate decline much faster than those of the r^{damage} rate, leading to a more conservative estimate and a wider interval for requirements R1 and R2.

Supplementary References

[1] Michael R. Benjamin, Henrik Schmidt, Paul M. Newman, and John J. Leonard. Autonomy for unmanned marine vehicles with MOOS-IvP. In Mae L. Seto, editor, *Marine Robot Autonomy*, pages 47–90. Springer, 2013.
